# Development of the ATAQ-IPF: a tool to assess quality of life in IPF

**DOI:** 10.1186/1477-7525-8-77

**Published:** 2010-07-31

**Authors:** Jeffrey J Swigris, Sandra R Wilson, Kathy E Green, David B Sprunger, Kevin K Brown, Frederick S Wamboldt

**Affiliations:** 1 Autoimmune Lung Center and Interstitial Lung Disease Program, National Jewish Health, 1400 Jackson Street, Denver, Colorado, 80206, USA; 2 Palo Alto Medical Foundation Research Institute, Palo Alto Medical Foundation, 795 El Camino Real, Palo Alto, California, 94301, USA; 3 Morgridge College of Education, University of Denver, 2199 S University Blvd, Denver, Colorado, 80210, USA; 4 Division of Psychosocial Medicine, National Jewish Health, 1400 Jackson Street, Denver, Colorado, 80206, USA

## Abstract

**Background:**

There is no disease-specific instrument to assess health-related quality of life (HRQL) in patients with idiopathic pulmonary fibrosis (IPF).

**Methods:**

Patients' perspectives were collected to develop domains and items for an IPF-specific HRQL instrument. We used item variance and Rasch analysis to construct the ATAQ-IPF (A Tool to Assess Quality of life in IPF).

**Results:**

The ATAQ-IPF version 1 is composed of 74 items comprising 13 domains. All items fit the Rasch model. Domains and the total instrument possess acceptable psychometric characteristics for a multidimensional questionnaire. The pattern of correlations between ATAQ-IPF scores and physiologic variables known to be important in IPF, along with significant differences in ATAQ-IPF scores between subjects using versus those not using supplemental oxygen, support its validity.

**Conclusions:**

Patient-centered and careful statistical methodologies were used to construct the ATAQ-IPF version 1, an IPF-specific HRQL instrument. Simple summation scoring is used to derive individual domain scores as well as a total score. Results support the validity of the ATAQ-IPF, and future studies will build on that validity.

## Introduction

Patient reported outcomes (PRO), such as quality of life (QOL) or health-related QOL (HRQL), are commonly used endpoints in clinical studies and therapeutic trials in patients with pulmonary diseases. Instruments that assess PRO focus on the perceptions of patients with the condition of interest; as such, they generate meaningful data on disease effects not captured by other outcome measures.

HRQL instruments are generic or disease-specific. The merit of disease-specific instruments is that they contain only items pertinent to patients with the disease of interest. Because of this, disease-specific instruments tend to be more responsive than generic instruments to underlying change. Disease-specific HRQL instruments have been developed for a number of pulmonary conditions, including chronic obstructive pulmonary disease[1-3] and asthma,[4,5] but not for idiopathic pulmonary fibrosis (IPF).

IPF is a progressive, fibrosing, parenchymal lung disease[6] with distinctive pathophysiological processes. IPF has no reliably effective therapy, and survival rates are worse than for many cancers [7]. In people with IPF, dyspnea limits physical activity, and hypoxemia ultimately develops, requiring patients to use supplemental oxygen. Given these discomforting aspects and the poor survival rates, it is not surprising that generic HRQL in patients with IPF is impaired [8,9]. Because IPF lacks a cure, there is a great deal of interest in maintaining or improving HRQL, so patients can live with acceptable QOL for however long they survive. Without a disease-specific instrument, there will continue to be uncertainty regarding whether relevant aspects and effects of the disease are being measured adequately and whether drug therapies, or other interventions, have a net beneficial or adverse impact on HRQL. In this manuscript, we report on the development an IPF-specific HRQL instrument called the ATAQ-IPF (A Tool to Assess QOL in IPF) version 1.

## Methods

### Questionnaire Development

#### Phase I: Item Development

Development of the ATAQ-IPF began with the conduct of three focus groups and five in-depth interviews with individual IPF patients, through which we conceptualized a framework for describing HRQL in IPF. Details of this step were reported previously [10]. We used themes and whenever possible, exact phrases spoken by focus group members or interviewees to develop domains and a pool of over 200 total items. In two additional focus groups, each with eight IPF patients, we reviewed domains (derived from themes) and items to ensure appropriate wording and coverage and to make revisions if necessary. Reordering and renaming of the original 12 yielded 14 domains: Cough, Dyspnea, Forethought, Sleep, Mortality, Exhaustion, Emotional Well-being, Spirituality, Social Participation, Finances, Independence, Sexual Health, Relationships, and Therapies. At this stage, the pool consisted of 207 items. All items employed a five-point Likert response format.

#### Phase II: Domain and Response Category Refinement and Item Reduction

Next, we enrolled 95 subjects with IPF (89 from the Interstitial Lung Disease (ILD) clinic at National Jewish Health and 6 from the ILD clinic at the University of Pennsylvania) who responded to the 207-item pool. IPF was diagnosed by multi-disciplinary consensus, according to internationally accepted guidelines [6]. We sequentially applied a selection criterion (based on response variance) and Rasch analysis to pare down items. First, items were retained if the sum of the proportion of respondents affirming response options (1) "Strongly disagree" or (2) "Disagree somewhat" was ≥ 25% and options (4) "Agree somewhat" or (5) "Strongly agree" was ≥ 25% (i.e., 1 + 2 ≥ 25% and 4 + 5 ≥ 25%); other items were eliminated.

Next, separate Rasch analyses[11] were performed on clusters of retained items within each of the 14 individual domains and then on the resultant item pool in its entirety after item elimination at the domain level. In Rasch analysis, a mathematical model is generated to describe the relationship between respondents and the items that operationalize a construct (or trait). For our purposes, for the analyses performed on the individual domains, the constructs are implied by the domain names (e.g., cough, dyspnea, exhaustion, etc.), and for the analysis of the entire item pool after item elimination, the over-arching construct is impairment in HRQL.

The Rasch model generates two estimates, called person location (or logit) and item location (or logit), which are nonlinear (log odds) transformations of raw scores. The likelihood of higher scores (i.e., person logit) increases as patients have more of the trait; thus, for our purposes, respondents with higher scores have greater impairments in the constructs tapped by the individual domains or in global HRQL. By placing person and item logits along opposite sides of a vertical line, in what is called an item map, Rasch analysis reveals how well items target the population under study. For dichotomous items (not the case for the ATAQ-IPF), when person and item logits are equal (i.e., directly across from each other on the item map), the person has a 50% probability of affirming the item. A respondent with more of a trait--thus, greater person logit--would be expected to affirm any item with a logit less than his person logit. For polytomous items, like those from the ATAQ-IPF, the analysis generates logit positions at the transitions between any adjacent response options (e.g., where the likelihood of responding "Strongly agree" is greater than the likelihood of responding to the adjacent option "Agree somewhat" and so-on). If requirements of the Rasch model are met, the scale (here, this holds for the individual domains and for the instrument in its entirety) will have additive measurement properties, or "behave like a ruler" [12].

There are no absolute criteria, but perhaps the most commonly used measure of item fit to the Rasch model--and the one we employed--is the infit mean square statistic. We identified items that both fit the Rasch model (infit mean square statistic 0.5-1.5 is considered useful for measurement[13]) and adequately covered the range of person locations according to the item map. Because having multiple items at the same logit position does not substantially add to a questionnaire's capacity to distinguish respondents with differing levels of the trait under study, we deleted excess items clustered at the same logit position. In sum, for paring down items, we followed these steps: 1) examination of item response variance and deletion of items that did not meet the criterion; 2) Rasch analysis on clusters of items within each domain and deletion of poor-fitting or redundant items; and 3) Rasch analysis of all retained items to ensure fit to the Rasch model and to generate statistics for the instrument as a whole.

### Psychometric Testing of ATAQ-IPF items

We used Pearson correlation coefficients to examine associations between domain scores and between scores for each domain and all other items in aggregate (exclusive of the domain under study). We assessed internal consistency reliability of each domain and the entire instrument with Cronbach's coefficient alpha [14]. Experts suggest alpha should be 0.7-0.9 for subscales of a multi-dimensional questionnaire,[15] with goal values of 0.9 for individual placement and ≥ 0.7 for research purposes [16]. Rasch model reliability was assessed by using the reliability of the person separation index, similar in its interpretation to Cronbach's coefficient alpha.

### ATAQ-IPF scores and their associations with clinical measures

Simple summation scoring is used to produce domain scores and a total score (range 74-370). Higher scores correspond to greater impairment.

On the day the questionnaire was completed, each subject performed pulmonary function tests (PFT) and a six-minute walk test (6MWT). PFT were performed according to American Thoracic Society standards, and results are reported as percentages of the predicted values (e.g., FVC% or DLCO%) [17,18]. The 6MWT was conducted as described previously, and distance walked (6MWD) was recorded [19]. Variables were tested for normality by using the Shapiro-Wilk test. Pearson (for normally distributed variables) or Spearman (for non-normally distributed variables) correlation was used to test the null hypothesis of no association between FVC%, DLCO%, or 6MWD and ATAQ-IPF domain and total scores. We also used multivariable linear regression to examine the relationship between the ATAQ-IPF total score and both FVC% and DLCO%. We used t tests (for normally distributed variables) or the Wilcoxon rank-sum test (for non-normally distributed variables) to compare mean ATAQ-IPF scores between subjects using versus not using supplemental oxygen. We hypothesized scores would be higher (more impairment in HRQL) for subjects requiring supplemental oxygen.

### Statistical Issues

Winsteps version 3.69.1.14 http://www.Winsteps.com was used to perform the Rasch analyses. SAS version 9.2 (SAS, Inc.; Cary, NC) was used to run all other statistics. We considered *p *< 0.05 as statistically significant. This project complied with the Helsinki Declaration. Each subject signed an informed consent, and the study protocol was approved by the Institutional Review Boards of the University of Pennsylvania and National Jewish Health.

## Results

### Baseline characteristics

Table 1 displays baseline demographic and disease parameters (including ATAQ-IPF scores) for the study sample. The mean time from diagnosis to questionnaire completion was 2.9 years. Just over 60% of the sample used supplemental oxygen, and mean physiology values suggested moderately severe IPF.

**Table 1:** Baseline Characteristics of Subjects

Male, %	82
	
Ethnicity, %	
Caucasian	94
Black	1
Other	5
	
Age yrs	69.3 (7.6)
	
Smoking status, %	
Past	64
Never	36
	
Had surgical biopsy, %	56
	
Time since diagnosis, yrs	2.9 (2.8)
	
Using supplemental O2, %	
Not at all	39
Exertion and sleep	31
Continuous	30
	
FVC%	65 (17)
DLCO%	39 (15)
6MWD, feet	1147 (441)
	
Taking IPF medications, %	
Prednisone	24
Azathioprine	14
N-acetyl cysteine	24
	
Carries a diagnosis of ___, %	
Emphysema (by HRCT)	15
PH by echocardiogram	31
Stable CAD	24
	
ATAQ-IPF scores:	Raw T
Cough	16 (7)
Dyspnea	19 (6)
Forethought	14 (6)
Sleep	16 (5)
Mortality	17 (5)
Exhaustion	15 (5)
Emotional Well-Being	20 (6)
Social Participation	15 (5)
Finances	17 (7)
Independence	14 (5)
Sexual Health	15 (6)
Relationships	17 (4)
Therapies	16 (4)
Total	210 (46)

Data presented as % or mean (standard deviation); O2 = oxygen; FVC% = percent predicted forced vital capacity; DLCO% = percent predicted diffusing capacity of the lung for carbon monoxide; COPD = chronic obstructive pulmonary disease; HRCT = high-resolution computed tomography scan; PH = pulmonary hypertension; CAD = coronary artery disease

### Item reduction

**Figure 1:**
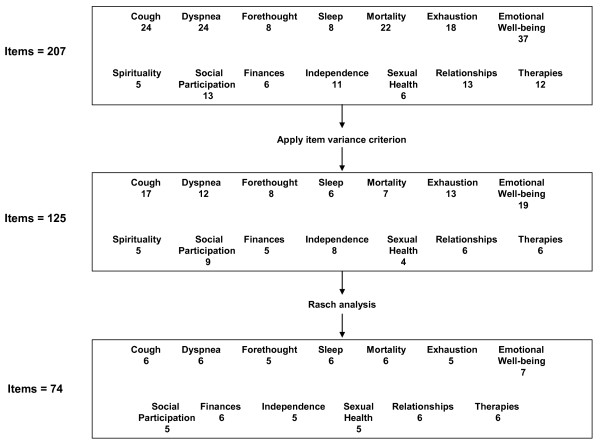
**Sequence of item reduction**.

**Figure 2:**
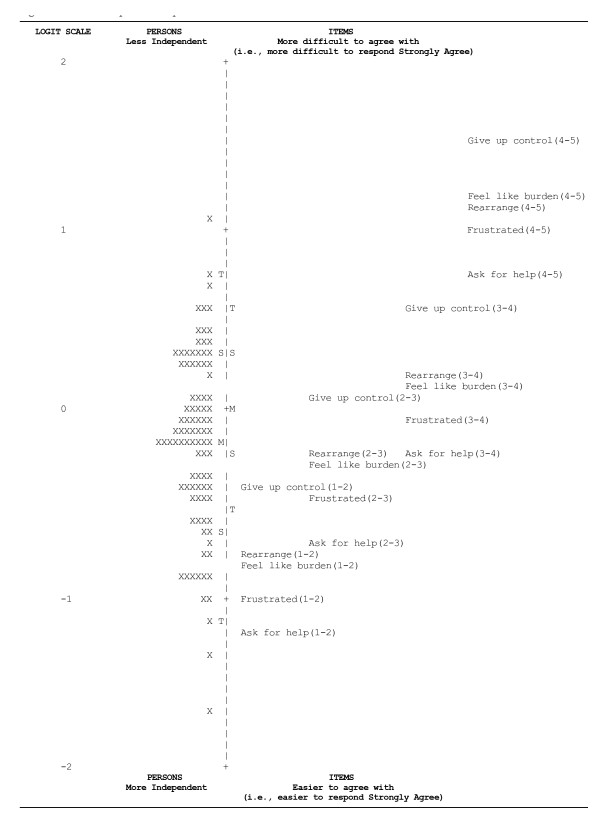
**Item map for Independence domain**. X = one subject; M = mean; S = one standard deviation from mean; T = two standard deviations from mean. The item positions for the five items in the independence domain appear on the right of the vertical dashed line. The person positions appear on the left of the line. Recall the five response options: (1)"Strongly disagree" (2)"Disagree somewhat" (3)"Neither disagree nor agree" (4)"Agree somewhat" and (5)"Strongly agree." Each item appears four times at logit positions that mark transitions between adjacent response options. The numbers in parentheses connote the adjacent response options. Thus, consider "Ask for help(1-2)" at the lowest (easiest) location on the map: this is the location where the likelihood that a subject would respond (2)"Disagree somewhat" to this item becomes greater than the likelihood he would respond (1)"Strongly disagree" to this item. The most difficult item from this domain (located at the top of the map) is "Give up control." The map is designed such that mean item location (difficulty) is at 0 logits (notice the "M" on the right side of the vertical line). Mean person location (ability, indicated by the "M" on the left side of the vertical line) is lower on the vertical line (i.e., fewer logits) than the mean item difficulty, thus indicating that item difficulty is slightly greater than person ability.

After the final two focus groups, the questionnaire had 207 items. On average, 40 minutes were required to respond to those items. After implementing the selection criterion based on item variance, 91 items were dropped, leaving 125 items for the Rasch analyses (Figure 1). The Finances, Sexual Health, Relationships, and Therapies domains were left with fewer than six items after the selection criterion. To perform a robust Rasch analysis on each of these domains, we included all their candidate items, even though some did not meet the variance criterion. An example of an item map for the Independence domain is displayed in Figure 2.

Domain-total correlations were statistically significant for every domain except Therapies. On balance, internal consistency reliability of the domains and overall instrument was excellent, and Rasch model reliability of person separation was good (Table 2). All retained items fit the Rasch model. Because of poor fitting items, the Spirituality domain and its items were dropped from the questionnaire, leaving 13 domains for the ATAQ-IPF version 1.

**Table 2:** Results of psychometric and Rasch analyses for the domains of the ATAQ-IPF.

Domain	Items (N)	Domain-Total Correlation (p value)	Internal Consistency Reliability*	Rasch Model Reliability
Cough	6	0.38 (0.0002)	0.92	0.83
Dyspnea	6	0.71 (<0.0001)	0.87	0.83
Forethought	5	0.67 (<0.0001)	0.89	0.82
Sleep	6	0.46 (<0.0001)	0.67	0.71
Mortality	6	0.42 (<0.0001)	0.74	0.78
Exhaustion	5	0.72 (<0.0001)	0.79	0.8
Emotional Well-being	7	0.68 (<0.0001)	0.85	0.82
Social Participation	5	0.65 (<0.0001)	0.81	0.78
Finances	6	0.49 (<0.0001)	0.9	0.67
Independence	5	0.72 (<0.0001)	0.81	0.74
Sexual Health	5	0.48 (<0.0001)	0.81	0.74
Relationships	6	0.6 (<0.0001)	0.61	0.71
Therapies	6	0.19 (0.07)	0.74	0.51
Total	74	-	0.94	0.93

*Cronbach's coefficient alpha

### Correlations with lung function and functional status

We observed significant correlations between measures of pulmonary physiology or functional capacity and ATAQ-IPF domain or total scores (Table 3). FVC% and DLCO% were significantly correlated with eight and nine respectively of the 13 ATAQ-IPF domain scores evaluated, as well as with the ATAQ-IPF total score. The 6MWD was significantly correlated with five domain scores as well as the ATAQ-IPF total. In a linear regression model of the ATAQ-IPF total score that included FVC% and DLCO% as predictors, FVC% (estimate = -0.09, p = 0.78) was not an independent predictor of the ATAQ-IPF total; DLCO% was (estimate = -1.57, p < 0.0001). The R-square value for this model was 0.25.

**Table 3:** Correlations between pulmonary function or six-minute walk distance and ATAQ-IPF scores.

Domain	FVC%	DLCO%	6MWD
Cough	-0.26 p = 0.01	-0.19 p = 0.08	-0.004 p = 0.98
Dyspnea	-0.40 p < 0.0001	-0.52 p < 0.0001	-0.23 p = 0.09
Forethought	-0.37 p = 0.0003	-0.58 p < 0.0001	-0.35 p = 0.009
Sleep	-0.18 p = 0.07	-0.1 p = 0.38	-0.18 p = 0.18
Mortality	0.14 p = 0.19	-0.05 p = 0.65	0.05 p = 0.73
Exhaustion	-0.33 p = 0.001	-0.46 p < 0.0001	-0.16 p = 0.26
Emotional Well-being	-0.19 p = 0.06	-0.32 p = 0.003	-0.18 p = 0.17
Social Participation	-0.21 p = 0.04	-0.51 p < 0.0001	-0.33 p = 0.01
Finances	-0.001 p = 0.98	-0.18 p = 0.12	-0.08 p = 0.58
Independence	-0.32 p = 0.0015	-0.47 p < 0.0001	-0.39 p = 0.004
Sexual Health	-0.20 p = 0.04	-0.55 p < 0.0001	-0.41 p = 0.002
Relationships	-0.28 p = 0.006	-0.40 p = 0.0002	-0.40 p = 0.003
Therapies	0.07 p = 0.48	0.21 p = 0.05	0.29 p = 0.03
ATAQ Total	-0.29 p = 0.006	-0.52 p < 0.0001	-0.28 p = 0.04

FVC% = percentage of predicted value for forced vital capacity;

DLCO%= percentage of predicted value for diffusing capacity of the lung for carbon monoxide; 6MWD = total distance walked during six-minute timed walk test; N = 95 for FVC, 82 for DLCO, and 54 for 6MWD

### Differences in ATAQ-IPF scores between subjects not using vs. those using supplemental oxygen

Nine domain scores (including Dyspnea and Exhaustion) and the ATAQ-IPF total score were significantly greater for subjects who required supplemental oxygen than for subjects who did not use supplemental oxygen (Table 4).

**Table 4:** Comparison of ATAQ-IPF scores between subjects using vs. not using supplemental oxygen.

Domain	Not using supplemental O2 N = 37	Using supplemental O2 N = 58	P value
Cough	15.9 (7.5)	16.2 (7.3)	0.6
Dyspnea	16.8 (6.6)	20.8 (6.0)	0.003
Forethought	10.9 (5.5)	16.2 (5.3)	<0.0001
Sleep	15.4 (4.6)	16.5 (4.7)	0.3
Mortality	16.8 (4.5)	17.4 (5.3)	0.6
Exhaustion	12.9 (4.6)	16.1 (4.5)	0.002
Emotional Well-being	18.0 (5.5)	21.1 (6.6)	0.01
Social Participation	11.7 (4.4)	16.3 (5.0)	<0.0001
Finances	16.3 (7.1)	17.4 (6.3)	0.4
Independence	11.6 (4.8)	15.8 (5.2)	0.0005
Sexual Health	12.7 (7.3)	16.3 (4.6)	0.0008
Relationships	15.2 (4.0)	18.3 (4.3)	0.0007
Therapies	16.9 (4.2)	15.1 (3.7)	0.04
ATAQ Total	191.0 (45.8)	223.1 (43.7)	0.001

## Discussion

We have developed the ATAQ-IPF version 1, an IPF-specific HRQL questionnaire. We used direct patient inquiry to generate an item pool, and we used rigorous statistical methods to reduce item numbers and construct an instrument that contains items tapping domains specifically relevant to patients with IPF.

In Phase I of item reduction, we deleted items with skewed response distributions--this serves the goal of maximizing the power of the ATAQ-IPF to discriminate between respondents with different degrees of HRQL impairment--and reduced item numbers by nearly half. We subjected the remaining items (in their domains and in aggregate) to Rasch analysis. The retained items--by virtue of fitting the Rasch model, like all items that fit the Rasch model--are guaranteed to have the same measurement characteristics as concrete physical measures (e.g., length or weight). Thus, by incorporating Rasch analysis into the development of the ATAQ-IPF, unlike other HRQL questionnaires for which Rasch methodology was not used, we can be confident that it adheres to the basic tenet of arithmetic: 'one more unit means the same amount extra, no matter how much we already have' [20]. So, an increase of one point for an ATAQ-IPF domain or total score means the same thing whether a respondent has severely impaired or near-normal HRQL. This linearity that the Rasch model constructs differs from the assumed linearity of classical test theory and much of item response theory--methodologies used to develop the majority of HRQL instruments [21].

By running Rasch analyses on clusters of items formulating each domain, we were able to pare down items in a systematic fashion. By dropping poor-fitting items, or certain ones from groups with identical logit positions (that only serve to make the questionnaire longer and not necessarily enhance the ATAQ-IPF's power to discriminate between respondents whose status changes over time), we were able to shorten the length of each domain.

The detailed and carefully executed item reduction techniques we used have not been implemented in the development of many other HRQL instruments. Generating content for the ATAQ-IPF, by directly capturing patients' perspectives and using them to build the framework (and specific items) of the questionnaire, ensure its content validity. Involving IPF patients in the development process ensures that all relevant themes and effects are tapped. It is the incorporation of such perspectives that makes the ATAQ-IPF uniquely applicable to IPF patients and not necessarily to patients with other forms of lung disease. Further, including only items that fit the Rasch model guarantees each of the ATAQ-IPF's scales (domain and total) maintain their additive properties. To our knowledge, only one other investigator has used this type of approach in the development of respiratory disease-specific HRQL instruments [2,3].

Psychometric testing revealed that domains and the overall instrument possess excellent internal consistency reliability [16]. Domain-total correlations confirmed that each domain measures some aspect of the same underlying construct--HRQL--and that each contributes information about HRQL unique from the aggregate contribution of the other items. The ATAQ-IPF, then, functions like an arithmetic test that has individual sections that assess addition, subtraction, multiplication, and division: the test score portrays overall arithmetic ability but the sections can point to areas in which a student might excel or need additional instruction. Likewise, the ATAQ-IPF overall scores serves as a measure of global HRQL, and the domain scores can be used to examine more closely the nature of the impact of an intervention on HRQL.

The significant correlations between domain scores and FVC%, DLCO%, and 6MWD showed that ATAQ-IPF scores are related to--but also yield their own unique information from--clinically meaningful, commonly used measures of IPF severity. Results from the linear regression analysis add more weight: in a model that controlled for arguably the two most important physiologic measures used to assess IPF patients (FVC% and DLCO%), those measures combined to explain only 25% of the variability (R-square = 0.25) in the ATAQ-IPF total score. Thus, there are factors not captured by these physiologic measures that contribute to HRQL in patients with IPF. Interestingly, there was moderately strong correlation between DLCO% and the Social Participation, Independence, and Sexual Health domains, and there were significant correlations between 6MWD and these domains as well as with the Relationships domain. These results indicate that gas exchange and functional capacity influence more than simply physical well-being, and they underscore the importance of extending HRQL measures to include such domains in patients with IPF.

Investigators commonly view significant associations between HRQL scores and clinical measures of disease severity or functional status as evidence for the validity of an instrument; however, the importance of such associations is primarily in understanding which manifestations of a disease have the greatest effects on HRQL--they are much less relevant to validity. So, although such correlations in this study confirmed our hypotheses that HRQL would be related to IPF severity (as measured by these physiologic variables), the validity of the ATAQ-IPF (or any other instrument) is best judged over time on three other terms: 1) its content--whether it covers all the relevant dimensions on which individuals evaluate their HRQL, or at least those that might be affected by the disease in question; 2) whether items require respondents to indicate the extent to which their QOL (on the various domains) is compromised by their disease; and 3) whether resulting scores are reliable, sensitive, and responsive to change. The ATAQ-IPF certainly meets terms 1 and 2, and further investigation will determine term 3. As with any HRQL questionnaire, validity is not achieved (or even determined) in a single study--it is built. It is only through observing the performance of a questionnaire in multiple studies over time that we can confidently say that it measures what it was intended to measure. That said, the results of the analysis in which we examined differences in ATAQ-IPF scores between subjects not using and those using supplemental oxygen support the validity of the ATAQ-IPF: subjects using supplemental oxygen had more dyspnea and exhaustion, less independence, required more forethought, and had greater impairments in emotional well-being, social participation, sexual health, relationships, and overall HRQL (according to the ATAQ-IPF total) than subjects not using supplemental oxygen.

Although 74 items comprise version 1 of the ATAQ-IPF, this number of items enables it to tap myriad important constructs and to report scores at the domain level. Whether item number can be reduced further, without unacceptable loss of content or reliability, requires additional investigation. Moving forward, we will use the ATAQ-IPF as a secondary outcome measure in a longitudinal study, and we invite other investigators to use the ATAQ-IPF version 1 in their studies as well.

## Conclusion

In sum, we have developed an IPF-specific instrument to measure HRQL. We used patients' views to generate themes and items and then systematically implemented statistical techniques to pare down item number. Items fit the Rasch model, and internal consistency supported reporting of domain and total scores. In future studies, data will be gathered to help further support the ATAQ-IPF's validity in IPF and to determine if it might be useful in other forms of interstitial lung disease.

## Competing interests

JJS is supported in part by a Career Development Award from the NIH (K23 HL092227). The authors declare that they have no competing interests

## Authors' contributions

Study conceptualization: JJS, SW. Data collection: JJS, DS, KB. Data analysis: JJS, SW, KG, FW. Writing and final approval of manuscript: JJS, SW, KG, DS, KB, FW.
